# Clinical significance of Neutrophil gelatinase-associated lipocalin(NGAL) expression in primary rectal cancer

**DOI:** 10.1186/1471-2407-9-134

**Published:** 2009-05-06

**Authors:** Xiu-Feng Zhang, Ying Zhang, Xiao-Hua Zhang, Su-Mei Zhou, Guan-Gen Yang, Ou-Chen Wang, Gui-Long Guo, Gao-Yi Yang, Xiao-Qu Hu

**Affiliations:** 1 Department of Oncological Surgery, the First Affiliated Hospital of Wenzhou Medical College, Wenzhou, 325000, PR China; 2 Department of Ultrasound, the Red Cross Hospital of Hangzhou, Hangzhou, 310003, PR China; 3 Department of Colorectal Surgery, the Third People's Hospital of Hangzhou, Hangzhou, 310009, PR China

## Abstract

**Background:**

Emerging evidence has demonstrated that Neutrophil gelatinase-associated lipocalin (NGAL) is up-regulated in multiple malignancies, including oesophagus cancer, and plays a critical role in tumorigenesis and progression. However, till now, little is known about the role of NGAL in human rectal cancer. Its association with clinicopathologic characteristics and expression of MMP-9, one of its target genes, has not been reported systematically in rectal cancer. Therefore, to further determine the potential involvement of NGAL in rectal cancer, we have evaluated the expression level of NGAL mRNA by real time RT-PCR, and further elucidated the correlation of NGAL mRNA expression with clinicopathologic features and MMP-9 in rectal cancer.

**Methods:**

100 paired samples of rectal cancer and adjacent normal tissues obtained from inpatients undergoing surgical operation were allocated into two groups (cancer group and control group). The mRNA expression of NGAL and MMP-9 was determined by real-time RT-PCR. The association between their expression and clinicopathological characteristics of rectal cancer were analysised.

**Results:**

Among the 100 rectal cancers, 69 cases of NGAL mRNA up-regulation were observed. NGAL mRNA up-regulation was positively correlated with MMP-9 (*r*_s _= 0.393, *p *< 0.001). In rectal cancer, NGAL mRNA overexpression was significantly associated with depth of invasion (*p *= 0.028), lymph node metastasis (*p *= 0.009), venous involvement (*p *= 0.023) and advanced pTNM stage (*p *= 0.011).

**Conclusion:**

In human rectal cancer, NGAL mRNA expression was elevated. NGAL mRNA up-regulation was correlated significantly with tumor progression and MMP-9 mRNA overexpression in rectal cancer, suggesting a more aggressive phenotype. NGAL could be used for rectal cancer characterization.

## Background

Colorectal cancer (CRC) is one of the common gastrointestinal cancers worldwide and the second leading cause of cancer-related deaths in Europe[1]. In the United States, colorectal cancer was the third most common cause of cancer related death in 2007[2]. About 30% to 60% of patients with colorectal cancer undergoing primary surgery with curative intention still die from metastatic disease[3]. Compared to colon cancer, rectal cancer, with worse prognosis, is mainly attributed to higher advanced cancer resulting from progression and metastasis of the tumor, which are two of the most important factors in determining the prognosis of patients with rectal cancers[4]. The early diagnosis of rectal cancer is difficult owing to the late presentation of symptom. The scarcity of early biomarkers has considerably hindered our ability to launch preventive measures for this malignancy in a timely manner. So it is important to find superior tumor markers implicated in the progression and metastasis of human rectal cancer.

NGAL, a 24-kDa glycoprotein, also known as lipocalin-2, belongs to the lipocalin protein family. The rat NGAL homologue, neu-related lipocalin (NRL), was first identified as a gene whose expression was specifically induced in HER-2/neu oncogene-induced rat mammary carcinomas[5]. NGAL exists as monomer (25 kDa), homodimer (46 kDa), and disulfide-linked heterodimer with Matrix Metalloproteinase-9 (MMP-9,135 kDa)[6,7]. As a member of the MMP family, MMP-9 has been implicated to cancer progression by degradation of the molecular components of the basement membrane and extracellular matrix, liberating vascular endothelial cell growth factor (VEGF) from the extracellular matrix and therefore enabling angiogenesis, invasion and distant metastasis [8-10].

Growing evidence has demonstrated that NGAL can form a complex with MMP-9 and improves MMP-9 expression, prevents its degradation, causing increased MMP-9 enzymatic activity, thereby favoring invasive and metastatic potential of cancer cells in vivo and in vitro [11-13].

Elevated NGAL expression has also been detected in a variety of malignancies and involved in the invasion and progression of tumors, including breast cancer, esophageal carcinoma and gastric cancer et al [13-15].

Up to now, however, little is known about the role of NGAL in human rectal cancer. Its association with clinicopathologic characteristics and expression of MMP-9, one of its target genes, in rectal cancer has not been reported systematically. Therefore, to further determine the potential involvement of NGAL in rectal cancer, we detected mRNA expression of NGAL and MMP-9 in matched rectal samples by real time RT-PCR, and further evaluated the correlation between NGAL and MMP-9 as well as clinicopathological features in human rectal cancer.

## Methods

### Patients and specimens

100 paired samples of rectal cancer and adjacent normal tissues were obtained from inpatients undergoing surgical operation from January 2006 to September 2008 at the Department of Oncological Surgery, the First Affiliated Hospital of Wenzhou Medical College. These inpatients did not receive any chemotherapy and radiotherapy before operation. This study was approved by the institutional review board and all patients provided written informed consent to participate. The rectal cancer and paired normal rectal tissues were allocated to a cancer and a control group respectively. The adjacent normal tissues were distant from the corresponding rectal cancer tissues more than 5 cm. The samples of rectal cancer and adjacent normal tissues were away from the region of obvious inflammation and necrosis. The immunohistochemistry Staining was routinely performed by the Department of Pathology of the First Affiliated Hospital of Wenzhou Medical College in order to exclude the cases with obvious inflammation and necrosis response, and to reduce the potential interference by neutrophils. All dissected specimens were cut into 5-mm cubic blocks, snap frozen in liquid nitrogen immediately and stored at -80°C. Tumor grade of differentiation was defined according to the WHO criteria[16]. The clinical and pathological stages were defined according to pTNM stage. The histopathological examination was conducted by the Department of Pathology of the First Affiliated Hospital of Wenzhou Medical College. As shown in table 1, specific primers and probes for NGAL gene, MMP-9 gene and glyceraldehydes-3 phosphate (GAPDH) gene (reference gene) were designed based on sequence data from the Ensembl database http://www.ensembl.org. Primers and probe were placed at the junction between 2 exons. All primers and probes were synthesized by Takara Biotechnology Co., Ltd (Dalian, China).

**Table 1:** Primers and probes

	Sequence
***NGAL***	
Forward primer	5'-ATGACATGAACCTGCTCGATA-3'
Reverse primer	5'-TCATAGTCGTTCATTATCTTC-3'
Probe	5'-FAM- CCTGAAAAGAGTCTCTGCCCA-TAMRA-3'
***MMP-9***	
Forward primer	5'-CTGAAATGACGTCCCTAAGT-3'
Reverse primer	5'-AGGAGGTCTCACTATCTGGAT-3'
Probe	5'-FAM- TCTGACATGCCTCGAGGACCT-TAMRA-3'
***GAPDH***	
Forward primer	5'-CCTCAAGATCATCAGCAAT-3'
Reverse primer	5'-CCATCCACAGTCTTCTGGGT-3'
Probe	5'-FAM- ACCACAGTCCATGCCATCAC-TAMRA-3'

### Real-time RT-PCR

Real-time RT-PCR was performed using a relative quantification protocol on an iCycler iQ System (Bio-Rad, Hercules, Calif., USA). The optimal conditions for the amplification of NGAL consisted of 2 min at 94°C followed by 40 cycles for 30 s at 94°C, 30 s at 51°C and 30 s at 71°C. The optimal conditions for the amplification of MMP-9 consisted of 2 min at 94°C followed by 40 cycles for 30 s at 94°C, 30 s at 56°C and 30 s at 71°C. In addition, a no-template control (double-distilled H_2_O control) was included for each master mix. All samples were amplified simultaneously in triplicate in a one-assay run. The amplified products were resolved by electrophoresis in 2.0% agarose gel and visualized by ethidium bromide staining. GAPDH was performed simultaneously as a standard internal control. The relative expression ratio (R) and real-time RT-PCR efficiency (E) of samples were determined by the following equations, as described in previous study[17,18].

[17]

[18]

The CT value is the cycle number at which the fluorescence signal crosses the threshold, and ΔCT is the CT deviation of the control minus the sample of the target or reference gene transcript. A ratio > 1 represented NGAL or MMP-9 mRNA up-regulation in rectal cancer relative to the control.

### Statistical analysis

SPSS 13.0 software for Windows (SPSS Inc, USA) was used for statistical analysis. The relative expression analysis and group-wise comparison of the target gene was performed by the Relative Expression Software Tool (REST, available at http://www.gene-quantification.com)[19]. Continuous variables were expressed as ( ± *s*). The expression of NGAL mRNA was assessed for associations with clinicopathological characteristics using Student's t-test for age and the χ^2 ^test for the remaining parameters. Finally, the correlation between NGAL and MMP-9 was evaluated by calculating Spearman's correlation coefficient. *p *< 0.05 was considered statistically significant.

## Results

### NGAL and MMP-9 mRNA Expression in Rectal Cancer and Paired Normal Rectal Tissues

The mRNA expression of NGAL and MMP-9 was examined in 100 rectal cancers and paired normal rectal tissues. Real-time RT-PCR amplification efficiencies were calculated from the given slopes in the FTC-2000 software (Fengling Co., Shanghai, China) and REST, and the amplification efficiency in the 2 groups was 2. As described above, the relative ratio, R, is presented as the n-fold change in gene expression normalized to an endogenous reference gene and relative to the control. Therefore, a value of R > 1.0 was considered to represent over expression of NGAL and MMP-9 gene in the sample relative to the control.

**Figure 1:**
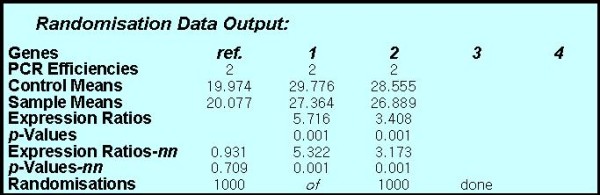
**Output of randomisation analysis in 2 groups by REST**. REST:Relative expression software tool. Genes ref.: GAPDH; Genes 1: NGAL; Genes 2: MMP-9 Sample means: Mean CT values for the rectal cancer tissues. Control means:Mean CT values for the adjacent normal tissues. Expression ratios-nn: Expression ratios not normalised. *p *values-nn: *p *values not normalised.

Among the 100 rectal cancer RNA samples tested, NGAL and MMP-9 over expression were shown in 69 and 63 cases respectively. Over expression of mRNA NGAL and MMP-9 was observed as follows: 52 cases both over expressed, 20 cases both not over expressed, 17 cases NGAL over expressed but MMP-9 not overexpressed,11 cases MMP-9 over expressed but NGAL not over expressed. The numeric results of the randomization test were put in the randomization box. REST analysis showed mRNA up-regulation of NGAL and MMP-9 in the sample group was remarkably different from that in the control group (*p *= 0.001), that is, it was up regulated in comparison to the control group (table 2, Figure 1).

**Table 2:** Output of randomisation analysis in 2 groups by REST

	GAPDH	NGAL	MMP-9
**Number**	100	100	100
**PCR efficiencies**	2	2	2
**Control means**	19.974	29.776	28.555
**Sample means**	20.077	27.364	26.889
**Expression ratios**		5.716	3.408
***p *values**		0.001	0.001
**Expression ratios-nn**	0.931	5.322	3.173
***p *values-nn**	0.709	0.001	0.001
**Randomisations**	1,000 of 1,000 done		

REST: Relative expression software tool.

Sample means: Mean CT values for the rectal cancer tissues.

Control means: Mean CT values for the adjacent normal tissues.

Expression ratios-nn: Expression ratios not normalised.

*p *values-nn: *p *values not normalised.

Randomisations: REST (Relative Expression Software Tool) is a standalone software tool to estimate up and down regulation for gene expression study in real-time PCR by using statistical randomization tests, while taking into account issues of reaction efficiency and reference gene normalisation. The randomization scenario is as follows: "if any perceived variation between samples and controls is due only to chance, then we could randomly swap values between the 2 groups and not see any greater difference than what we see between the initial groups."In our study, the hypothesis test performs 1,000 random reallocations of samples and controls between the groups, and counts the number of times the relative expression on the randomly assigned group is greater than the sample data.

### Relationship between NGAL mRNA up-regulation and Clinicopathological Features in Rectal Cancer

The relationship between NGAL mRNA up-regulation and clinicopathological characteristics was analyzed. NGAL mRNA up-regulation correlated significantly with depth of invasion (*p *= 0.028), lymph node metastasis (*p *= 0.009), venous involvement (*p *= 0.023) and advanced pTNM stage (*p *= 0.011). There was no correlation of NGAL mRNA with age, gender and grade (*p *> 0.05, table 3).

**Table 3:** Relationship between expression of NGAL mRNA and clinicopathological characteristics in rectal cancer

Variables/categories	NGAL expression		*P *value
		
	ratio ≤ 1	ratio > 1	
**Age, years**	63.38 ± 11.1	60.78 ± 13.1	NS
**Gender**			NS
Male	13	31	
Female	18	38	
**Grade**			NS
Well-differentiated	4	3	
Moderately differentiated	20	51	
Poorly differentiated	7	15	
**Depth of invasion**			0.028
pT1	5	2	
pT2	13	23	
pT3	12	33	
pT4	1	11	
**Lymph node metastasis**			0.009
Absent	17	19	
Present	14	50	
**Venous involvement**			0.023
Absent	26	42	
Present	5	27	
**pTNM stage**			0.011
pI	4	2	
pII	13	14	
pIII	13	44	
pIV	1	9	

ratio ≤ 1: The expression of NGAL mRNA was not up-regulated; ratio > 1: The expression of NGAL mRNA was up-regulated; NS:Not significant.

### Correlation between mRNA up-regulation of NGAL and MMP-9 in Rectal Cancer

In rectal cancers, the correlation coefficient between mRNA up-regulation of NGAL and MMP-9 was 0.393. NGAL mRNA up-regulation was positively correlated with MMP-9 (*p *< 0.001, table 4).

**Table 4:** Correlation between mRNA up-regulation of NGAL and MMP-9

MMP-9 expression	NGAL expression		*P*	*r*
			
	ratio ≤ 1	ratio > 1		
**ratio ≤ 1**	20	17	< 0.001	0.393
**ratio > 1**	11	52		

ratio ≤ 1: The expression of NGAL or MMP-9 mRNA was not up-regulated; ratio > 1: The expression of NGAL or MMP-9 mRNA was up-regulated; *r*: Correlation coefficient.

## Discussion

This is the first study to apply the real-time RT-PCR method to quantify the NGAL mRNA expression and to elucidate the correlation of NGAL mRNA expression with clinicopathologic features and MMP-9 in human rectal cancer. The present study observed that 69 cases (69%) among the 100 rectal cancers showed NGAL mRNA overexpression, a significantly elevated level of NGAL mRNA expression with real-time RT-PCR in rectal cancers compared with adjacent normal tissues, which is in agreement with the report of Nielsen et al. [20] Hypomethylation of the NGAL gene has been found both in pancreatic and mammary tumor cell lines, which may be responsible for its high expression levels [21]. Moreover, we demonstrated that NGAL mRNA up-regulation correlated significantly with MMP-9 and some important clinicopathological characteristics such as depth of invasion, lymph node metastasis, venous involvement and advanced pTNM stage (p < 0.05). Depth of invasion, lymph node metastasis and pTNM stage of rectal cancer have powerful predictive value to prognosis. Venous invasion by tumor has also been demonstrated to be a stage-independent adverse prognostic factor [22,23]. This suggests that NGAL not only involved in the progression of rectal cancer, but also could be a prognostic marker in rectal cancer.

Interestingly, unlike our results, Lee and colleagues[24] reported that overexpression of NGAL suppressed human colon cancer KM12SM cell to invade Matrigel in vitro, and liver metastasis of colon cancer cells injected directly into the spleens in an mice experimental animal model. These apparently diverse observations could be due to distinct functions of NGAL in different cell types, which is further supported by a previous study demonstrating that NGAL overexpression could suppress Ras-transformed murine breast cancer 4T1 cell invasion and lung metastasis in vivo [25]. However, ectopic expression of NGAL may significantly stimulates the growth of MCF-7 human breast cancer cells in vivo[12]. Furthermore, such an experimental metastasis study may also have limitations in reflecting the whole process of metastasis since the effects on primary malignancy growth before metastasis could not be determined. Therefore, future evaluation of NGAL with a spontaneous metastasis assay was essential so as to elucidate the context-specific regulation mechanism.

MMPs are believed to play a crucial role in colorectal cancer tumor growth and angiogenesis, thereby promoting invasion and metastasis. MMP-9 appears to be one of the most important since it is overexpressed in the majority of colorectal cancers [26,27]. Several lines of evidence in vitro or in vivo have suggested that NGAL plays an important role in the enhanced invasive potential of cancer cells by upregulating MMP-9 expression. Yan et al demonstrated that NGAL is capable of protecting MMP-9 from degradation in a dose-dependent manner and thereby preserving MMP-9 enzymatic activity in vitro system by employing recombinant human MMP-9 and NGAL, as well as in a cell culture system in which NGAL was overexpressed in MDA-MB-231 human breast carcinoma cells. [11]Transfection of an NGAL expression plasmid conferred the invasive phenotype and motility activities of a noninvasive human breast cancer cell line (MCF-7) accompanying an increase in MMP-9 expression[12]. Furthermore, down-regulation of NGAL expression in oesophageal squamous cell carcinoma (ESCC) cells could significantly suppress MMP-9 activity and the invasion of these cells in nude mice[28]. Recently, Frank et al have reported the clinical evidence for a protective role of NGAL against MMP-9 autodegradation in gastric cancer[13]. In present study, we also revealed a significant elevated expression of NGAL and MMP-9 mRNA levels in human rectal cancer and they all correlated well with tumor invasion and metastasis. Additionally, NGAL mRNA overexpression was positively correlated with MMP-9 mRNA up-regulation (rs = 0.393, *p *< 0.001). This suggests that the NGAL-MMP-9 axis could be a potent therapeutic target in rectal cancer patients. Therapeutic agents that inhibit the expression or function of NGAL or of its target genes may prove efficacious, or might complement agents that directly compromise the MMP-9 activities in the treatment of human rectal cancer. Studies are needed to further explore the mechanisms involved.

The spread of malignant tumor cells from a primary tumor to form metastases at distant sites is the most life-threatening complication of cancer and is responsible for the majority of deaths in affected individuals. When detected at early stages, the majority of CRC can be cured with high success rates, and there is a strong correlation between tumor progression at the moment of the diagnosis and survival rate[29]. Hence, a marker to identify early stage cancer would be immensely useful to improve survival. For this purpose, a number of screening tests have been developed. However the ideal screening test must be specific, sensitive and non-invasive. NGAL as a valuable biomarker for early diagnosis and monitoring relapse of malignancy has been increasing used. Fernandez et al have demonstrated that NGAL and MMP-9 complexes were identified in nearly 86.36% of urine sample from breast cancer patients whereas this molecule is undetectable in the urine of healthy controls, suggesting that urinary NGAL may represent a novel, non-invasive biomarker for tracking disease status and the effectiveness of anticancer therapy in NGAL positive breast carcinomas[12]. In addition, serum NGAL measurement by ELISA is fairly accurate in distinguishing pancreatic cancer from non-cancer cases and could be investigated as a marker of pancreatic intraepithelial neoplasia (PIN) for early diagnosis of pancreatic cancer[30]. Furthermore, urinary NGAL and MMP-9 are useful predictors of the presence of brain tumors and may provide a basis for a novel, non-invasive method to identify new brain tumors and monitor known tumors after treatment[31]. Recently, Lim and colleagues[32] have also demonstrated that NGAL involved in the progression of epithelial ovarian malignanciesmay and may be served as an early screening biomarker to monitor changes of benign to premalignant and malignant ovarian tumors. This raises the possibility that serum and urine NGAL measurement (or combination with other potential biomarkers such as MMP-9) could also be explored as a utility for detecting disease status, progression, and therapeutic efficacy through noninvasive surveillance in rectal cancers, which deserves our attention.

## Conclusion

Till now, little is known about the potential involvement of NGAL in human rectal cancer. In present study, we detected the mRNA expression of NGAL and MMP-9 in human rectal cancer by real-time RT-PCR. Our research demonstrated that NGAL mRNA expression was elevated in human rectal cancer. NGAL mRNA up-regulation was correlated significantly with tumor progression and MMP-9 mRNA overexpression in rectal cancer, suggesting a more aggressive phenotype. NGAL detection may provide valuable information for rectal cancer characterization and identification of a subset of patients requiring more aggressive adjuvant therapy. As a secreted molecule, NGAL may serve as an attractive therapeutic target. Further studies are needed to elucidate the precise mechanisms of NGAL implicated in cancer progression and its potential utility in rectal cancer treatment.

## Competing interests

The authors declare that they have no competing interests.

## Authors' contributions

XFZ and SMZ carried out the experiments and drafted the manuscript. XHZ and GGY has been involved in revising the manuscript critically for important intellectual content. YZ participated in the design of the study and performed the statistical analysis. OCW participated in collecting the samples. GLG, GYY and XQH has been involved in designing the study and revising the manuscript. All authors read and approved the final manuscript.

## Pre-publication history

The pre-publication history for this paper can be accessed here:

http://www.biomedcentral.com/1471-2407/9/134/prepub
